# Are Dentists a Set of Enthusiasts?

**Published:** 1899-03

**Authors:** Frank W. Sage

**Affiliations:** Cincinnati, Ohio


### Are Dentists a Set of Enthusiasts?

BY FRANK W. SAGE, D.D.S., CINCINNATI, OHIO.

Read before the Ohio State Dental Society, December, 1898.

This question is asked in no caviling spirit. It is not intended to convey an impression of an uncomplimentary estimate of the profession, individually or collectively. A certain degree of enthusiasm is not only recognized as commendable in the dentist or in a body of dentists, but is held to be a requisite to progress. The inquiry which it is proposed to make is this: Does the history of the profession's researches within the last quarter of a century show consistent adherence to scientific principles and methods, in examining, testing, and adopting new methods and instrumentalities in dental practice? In other words, have we, in these matters named, reasoned legitimately from cause to effect (by analogy or otherwise), in trying to assign to them their proper value, or have we occasionally jumped to conclusions, accepting some one else's dictum, instead of exercising our own individual judgment?

Undoubtedly there has been exhibited a blind, unreasoning enthusiasm, of which we have been wholly unconscious. It is not because such enthusiasm leads inevitably to error that the fact requires to be expressly mentioned, but because it *may* lead to error.

The agony which *most frequently stirs up* a form of enthusiasm calculated to make the dentist or a body of dentists lose their heads takes on the form of a man. It is not usually—probably never—the man who has an axe to grind. (At least not as regards the dental assembly.) The individual *dentist's* nose may fail, but the nose of the body collective of dentists catches the faintest whiff of the wolf in sheep's clothing. No, the man who lays a spell upon us is probably innocent of any such design; is thoroughly sincere in his purpose of helping the profession, unselfish, and himself burning with enthusiasm. Add to these the quality of personal magnetism, and you have a man innocently and ignorantly dangerous to the last degree, when allowed free rein in a dental convention. Especially so if he be himself rather credulous, self-assertive and positive.

It may be of interest to pass in retrospect some of the "crazes," which within a few years have gained a more or less secure foothold in the profession. Not so many years ago the lacto-phosphate of lime treatment for exposed tooth-pulps was discussed in dental conventions far and near, stirring up a genuine sensation. It was proposed to apply a magma composed of phosphate of calcium and lactic acid to the pulp, protecting this with a temporary filling, the object being to induce the pulp to appropriate the pabulum thus offered, and in time transform it into a shield. Or perhaps it was a process of catalysis which was looked for, the writer is not sure.

As a sensation this was all very interesting. We can even afford to laugh a little at our credulity, at this late day. The question, however, arises, where were the men, all this time, who should have pointed out the physiological absurdity of presuming that the pulp would lend itself for a special occasion to perform a function wholly alien to its structure and organization? It would seem at least, that attention should have been called to the necessity of the pabulum's being exhibited through the usual channels whereby the appropriation and assimilation of inorganic matter is sought. But, no. Or if, at least, anything of the sort was urged, it was not effective as a plea in deterring many from experimenting along the line of treatment suggested.

Later came the implantation doctrine, founded on two assumptions, bold to the point of sublimity. The first was, that the periosteum of almost any old extracted tooth-root is capable of being revivified, provided it—the root—be restored to something like its original environments. The second was, that the artificial socket drilled in the maxilla would take on all necessary physiological functions for restoring the root inserted to pristine usefulness. The singular and highly interesting feature attending both this operation and the other we have named is, that a rather specious semblance of the genuine phenomenon sought for actually takes place many times; the tooth pulp becomes covered with a horny shield; the implanted tooth becomes firmly fixed in its new socket. But in both instances the effect is rather casual than the logical result of practically applying scientific theory. A quasi-scientific savor hangs about such operations as these, which may continue to beguile and befog men who will not take the trouble to do their own thinking long after more thoughtful men have given over the fallacy.

Set over against this too-credulous disposition in dentists, and strongly contrasting with it, is seen occasionally an unwillingness to accept on valid evidence what seems to be really a good thing. There is sometimes a total lack of enthusiasm, which is quite as remarkable and is often more unaccountable than over-enthusiasm at another time. Such, for instance, was the reception accorded the so-called Herbst method of filling teeth, a few years ago. Here was a method of filling not an experiment, but a thoroughly well-tried system, which promised and indeed fulfilled its promise of accomplishing gold-filling of cavities more readily, more expeditiously, with less effort on the operator's part, with less of an infliction on the patient, than the methods generally in vogue among the dentists of the United States. It was claimed that a tighter filling could be made by this method than with the mallet and pluggers, and in from three-fourths to one-half less time. The method was considered of enough importance to be fully described and illustrated in the *American System of Dentistry*, as the three large volumes issued about that time were called. But the method never came into general vogue. The singular thing about it all is that you can seldom get out of any man who formerly used it why he abandoned the Herbst method of filling.

The day of enthusiasts is not yet over. Just now it seems to be the new amalgam. We are unable to learn positively who vouches for its being the *only* amalgam; the story is that either the American Dental Association or a Tri-State Association stands sponsor for it. Nobody seems to know positively. But it is "in the air" that this amalgam has never had anything like an equal, and men are emptying their old amalgams into the ash-pan.

Now comes a belated statement that the formula for this amalgam is within a very slight fraction of being identical with that of another well-known amalgam, for years upon the market, devised by the well-esteemed author of the "New Departure" ideas.

So we go. Perhaps, after all, the inquiry we need to make is not, "are dentists a set of enthusiasts," but, are dentists a set of dupes?

DISCUSSION.

DR. F. A. HUNTER: *Mr. President and Gentlemen*:—I do not think I have been up long enough to say much. There seems to be two points that are always essential in opening the discussion upon any paper, the first is to compliment the essayist and the next is to differ with him. Unfortunately, in this case, I can only do the one and that is to compliment him, I will have to agree with him to a very great extent. This so-called enthusiasm of dentists, as he suggested, the tendency to run and follow a leader is not confined to our profession, it exists undoubtedly in the medical profession to a greater degree than ours; in fact, it seems to be one of the constituents of the human family, and I may say embraces the entire animal kingdom, if not the vegetable. Enthusiasm, of course, is good to a certain extent, but a few of the illustrations he has given are unfortunate. There was no enthusiasm in the introduction of the lacto-phosphate of lime treatment; I was there when the paper was read and it was condemned there by nearly everybody. I do not know that anybody thought anything of it. At that time we were all capping pulps more or less with all sorts of substances and materials and with universal success; we were so successful we finally thought nothing of it. There are some cases, of course, where pulps capped with any material will seem to live, but in the great majority of cases will die. Why, the great Dr. Atkinson reported success after success by the capping of pulps with the oxy-chloride of zinc, putting it directly in contact with the pulp and it was all right, simply because after the first paroxysms or pain the pulp died, and patient had no further trouble. Now, in regard to the Herbst method, I do not know anybody in our section except one doctor who ever took it up except, of course, Dr. Herbst. It was never a success. That is what we are here for, to try the remedies brought up and test their merits. So, in the general trend of the essayist's paper I will agree with him, we are a set of enthusiasts and we follow our leader, which is natural; if any one of us are enthusiastic over something he has discovered and consider it a good thing, we all want to try it. Again, of course, we run to extremes—that is human nature. I see on the program this year the word cataphoresis is omitted, two years ago we did not talk about anything else, everybody was having success with it, it was discovered the next year we were using the wrong pole of the battery and were doing no good with it whatever. Of course, we run wild for a time over these successes, and in many instances there are other things which account for these apparent triumphs. Then, again, is the tendency not to report failures and only successes, and in receiving same we take it for granted that all the rest are the same. In the medical profession some years ago after the introduction of KBr. potassium bromide, a remedy they went wild about, Dr. Black told me that on examining a prescription file of a prominent druggist in 1866, four years after the introduction, he found that during that one year more than three-fourths of the prescriptions filled contained potassium bromide. If that is not enthusiasm, I do not know. That feature to follow a leader is in the human family, and in the entire animal kingdom.

Dr. Callahan: Is it not oftentimes the case that enthusiasm will drown real virtue? We go from one extreme to another, this is the cause, and it seems to be a law in human nature. We condemned Herbst's method and passed it by. I should not like to discard it in certain applications, but not use it to the extent which was expected in the beginning. Cocaine was the only thing for a while, but there came a time when we decided it was not a good thing in all cases, so it is with a good many other remedies. So enthusiasm oftentimes drowns real virtue. As our time is limited, I would suggest that Dr. Smith close the discussion.

Dr. Taft: A word or two in reference to this matter of enthusiasm. Not to condemn it anywhere, unless, of course, it is the means of doing wrong. It is very frequently the case that only by the interest and enthusiastic advocation of persons about certain processes that they are introduced at all. It is true, too, that things that have not much of real value come to be used when persons come to be over-enthusiastic; but this is also necessary sometimes for the profession in order to get them to take hold of a matter at all. Any method or subject is not taken hold of with any good degree of perseverance unless somebody becomes enthusiastic, and then everybody thinks—well, there must be something in it, I will try it. Now that is one of the phases of the subject; another is this, there are comparatively few in the profession who are able to judge in reference to this subject, so we make mistakes, those that do take hold of the matter and test it in a kind of perfunctory way without getting into the real merits of the question, and without having a knowledge of the law or principles which governs the subject in question. We think because others have made success, therefore, we ought to make as great success. A great many things which have been successful in the hands of one has not been so in others. It seems to me before running wild about anything, we should study all about its use and principles and qualities of materials, understand and study and know about them, compare them with other things and materials and endeavor to learn all about them, not only by mere experimentation, but study processes involved, its qualities, purposes, etc., and what we may expect in comparison with other things. All these points will enable us to reach a true conclusion in reference to anything of this kind. We should guard against our becoming too enthusiastic, not take it up before thorough experimentation and study over it. We know that many things which have been advocated as very good, prove to be utter failures. The point of greatest importance in this subject is this: do not become so enthusiastic over anything that we will use it to the disadvantage of those whom we try to serve. We have done this many a time, no doubt. Do not let it run away with our reasoning power. This is the point which ought to be guarded. It is true that a great many of the profession, think that because Dr. A. has accomplished a certain operation, if they see it done once they can go on and do it, but they cannot. We must have a familiarity with the process based upon experience enough to overcome all difficulty. A great many things which have been laid aside are very good when we become thoroughly familiar with them. A great majority of the processes require extensive experience to get out of them all the value there is in them, and many a time that is laid aside which, with judicious use, would have been of great value.

Dr. Smith (closing discussion): There is very little more to be said in closing the discussion. One point which suggests itself in defense of enthusiasm, and Dr. Taft calls attention to the point when he states there are a very few of the profession who can step forward and correct all the other members of the profession from their enthusiasm. But the question is, how many can assume this responsibility? The essayist does not mention the fact that time itself is a great eliminator of mistaken ideas, and perhaps in regard to the various points, oxy-chloride of zinc and lacto-phosphate treatment of tooth pulps, time itself has been the teacher which has corrected the mistakes the dentists were making and balanced their enthusiasm. Further than that there is nothing to say.

